# The ion channels and transporters gene expression profile indicates a shift in excitability and metabolisms during malignant progression of Follicular Lymphoma

**DOI:** 10.1038/s41598-019-44661-x

**Published:** 2019-06-13

**Authors:** Alberto Magi, Marika Masselli, Cesare Sala, Angela Guerriero, Pasquale Laise, Benedetta Puccini, Luigi Rigacci, Carla Breschi, Olivia Crociani, Serena Pillozzi, Annarosa Arcangeli

**Affiliations:** 1 Department of Experimental and Clinical Medicine, University of Florence, Florence, Italy; 2 Department of Information Engineering, University of Florence, Florence, Italy; 3 Hematology Department, Azienda Ospedaliero Universitaria Careggi AOUC, Florence, Italy; 4 Hemato-oncology unit, Ospedale San Jacopo, Pistoia, Italy; 5 Present Address: Department of Systems Biology, Columbia University, New York, New York USA

**Keywords:** B-cell lymphoma, Microarrays

## Abstract

The definition of the gene expression profile of genes encoding Ion Channels and Transporters (ICT-GEP) represents a novel and attracting aspect in cancer. We determined the ICT-GEP of Follicular Lymphoma (FL), and compared it with that of the more aggressive Diffuse Large B Cell Lymphoma (DLBCL). cDNA microarray data were collected both from patients enrolled for this study, and from public datasets. In FL the ICT-GEP indicated the overexpression of both the K^+^ channel encoding gene *KCNN4*, and *SLC2A1*, which encodes the Glut1 glucose transporter. *SLC2A1* turned out to represent the hub of a functional network, connecting channels and transporters in FL. Relapsed FL patients were characterised by 38 differentially expressed ICT genes, among which *ATP9A*, *SLC2A1* and *KCNN4* were under-expressed, indicating a down-regulation of both excitability and glycolysis. A completely different profile of K^+^ channel encoding genes emerged in DLBCL accompanied by the over-expression of the fatty acid transporter-encoding gene *SLC27A1* as well as of the metabolism regulator *NCoR1*. This indicates a change in excitability and a shift towards an oxidative metabolism in DLBCL. Overall, the ICT-GEP may contribute to identifying novel lymphoma biomarkers related to excitability and metabolic pathways, with particular relevance for drug resistant, relapsed FL.

## Introduction

The two most common Non-Hodgkin lymphomas (NHLs) in developed countries are diffuse large B-cell lymphoma (DLBCL) and follicular lymphoma (FL), collectively accounting for nearly half of all NHLs^1,2^. FL is a low-grade germinal centre-derived tumour, characterised by a prolonged clinical course, with frequent relapses after treatment, 10% of which will not respond to chemo-immunotherapy. Furthermore, over the course of their illness, roughly half FL patients develop a more aggressive disease, which may eventually transform into a DLBCL^3,4^.

The identification of the gene expression profile (GEP) has been proven to be a powerful clinical tool in several types of cancers^5–8^ including lymphomas^9–11^. In these, the GEP has strongly contributed not only to the understanding of neoplastic B-cell biology but also for the diagnostic and prognostic characterisation of the disease. The GEP of DLBCL is now well characterised and validated for clinical applications^7,8^. In particular, GEP studies accelerated our understanding of the molecular complexity of DLBCL, and led to the identification of two molecularly distinct forms of DLBCL with gene expression patterns indicative of different stages of B-cell differentiation, resembling either germinal center B-cells (GCB) or activated B-cells (ABC)^7^. The GEP of FL has also been intensely studied in the last ten years^10–14^, and several driver genes and signalling pathways involved in the pathogenesis and progression of the tumour have been identified^9–12^. It remains still unsolved whether the aggressiveness of transformed FL occurs from the prevalent growth of an existing subclone, or results from novel genetic/epigenetic hits in the neoplastic B cells, or even to changes in the immune cells present within the tumour microenvironment^13,14^.

Besides their undiscussed role in the regulation of many physiological functions in both excitable and not excitable cells, ion channels and transporters (ICTs) are emerging as novel cancer biomarkers^15,16^. Recently, an ICT-based signature has been produced for gliomas, breast and lung cancers^16–18^ and has been proposed as a novel tool to predict survival and clinical outcome. The expression and role of ion channels in lymphocytes, mostly T-cells, has been investigated since almost thirty years, leading to the uncontested role of different K^+^ and Ca^2+^ currents in the regulation of lymphocyte excitability, which in turn drives their activation^19,20^. Few studies have addressed the expression and role of ICTs in lymphoma, and all of them have led to the identification and functional characterization of single molecular entities^7,21–23^. Overall, no lymphoma ICT-GEP has been provided so far.

Based on these premises, the purpose of the present study was to determine the ICT-GEP of FL, to be compared with that of the more aggressive relapsed FL and DLBCL, with the aim to identifying different profiles related to disease progression and therefore their potential translational relevance. We studied both samples from a cohort of patients specifically enrolled for this study as well as public datasets.

## Results

### Analysis of differentially expressed (DE) genes in FL samples

We collected 54 consecutive diseased lymph node samples, 13 of which were diagnosed as FL and six as DLBCL. After RNA extraction, cDNA microarray analysis was applied only to those samples (11 FL and 2 DLBCL) whose RNA integrity number (RIN) was >6 (Supplementary Table S1). The clinico-pathological characteristics of the patients enrolled are shown in Table 1. FL samples displayed different histological grades, and only the subtype A of grade III was present. Besides the expected Bcl2 alterations^24^, all the samples showed Bcl6 over expression (indicated by the pathologists’ diagnosis and confirmed by microarray data, see Table 1 and Supplementary Table S2) and 6 out of 11 experienced disease relapse towards a similar FL disease. The two DLBCL patients belonged to IIIA and IVA stages, showed different response to therapy, and neither relapsed.

**Table 1:** Clinical characteristics of samples collected in our cohort.

Sample n.	Age	Sex	Diagnosis	Stage	Bcl2	Bcl6	Response	Relapse	Time to relapse (months)	Time of OS (months)
1	74	M	FL III° A	II	+/−	+	CR	No	—	111
12	46	M	FL I°	IIIA	+	+	CR	No	—	106
13	68	M	FL II°	IVB	+	+	PR	Yes	18	38
18	79	M	FL I°	IVA	+/−	+	CR	Yes	83	186
19	63	F	FL II°	IA	+	+	CR	No	—	101
21	52	M	FL III° A	IVA	+	+	CR	Yes	60	160
23	69	F	FL II°	IIIA	+/−	+	CR	Yes	83	179
26	52	M	FL II°	IVA	+	+	CR	Yes	15	99
28	64	F	FL II°	IA	+	+	CR	No	—	113
36	61	M	FL III°A	IVA	+	+	CR	No	—	93
38	61	F	FL III°A	IA	−/+	+	CR	Yes	23	115
14	60	F	DLBCL	IVA	+	+	CR	No	—	104
30	62	F	DLBCL	IIIA	+	+	PR	No	—	8

In the first column, “Sample number”, the progressive numbers associated to the enrollment of each patient are reported. In the fourth column “Diagnosis” is reported the histological grade relative to each patient according to the WHO classification. FL is classified in subgroups from grade I to III (A or B). In the sixth and seventh columns is reported the expression of Bcl2 and Bcl6 oncogenes according to the clinico-pathological diagnosis. IGH/BCL2 rearrangement, leading to the overexpression of BCL2 protein, is a relatively specific molecular marker of FL that has been used for diagnostic and monitoring purposes.

Abbreviations: M, male; F, female; FL, follicular lymphoma; CR, complete remission; PR, partial remission; OS, overall survival.

**Figure 1:**
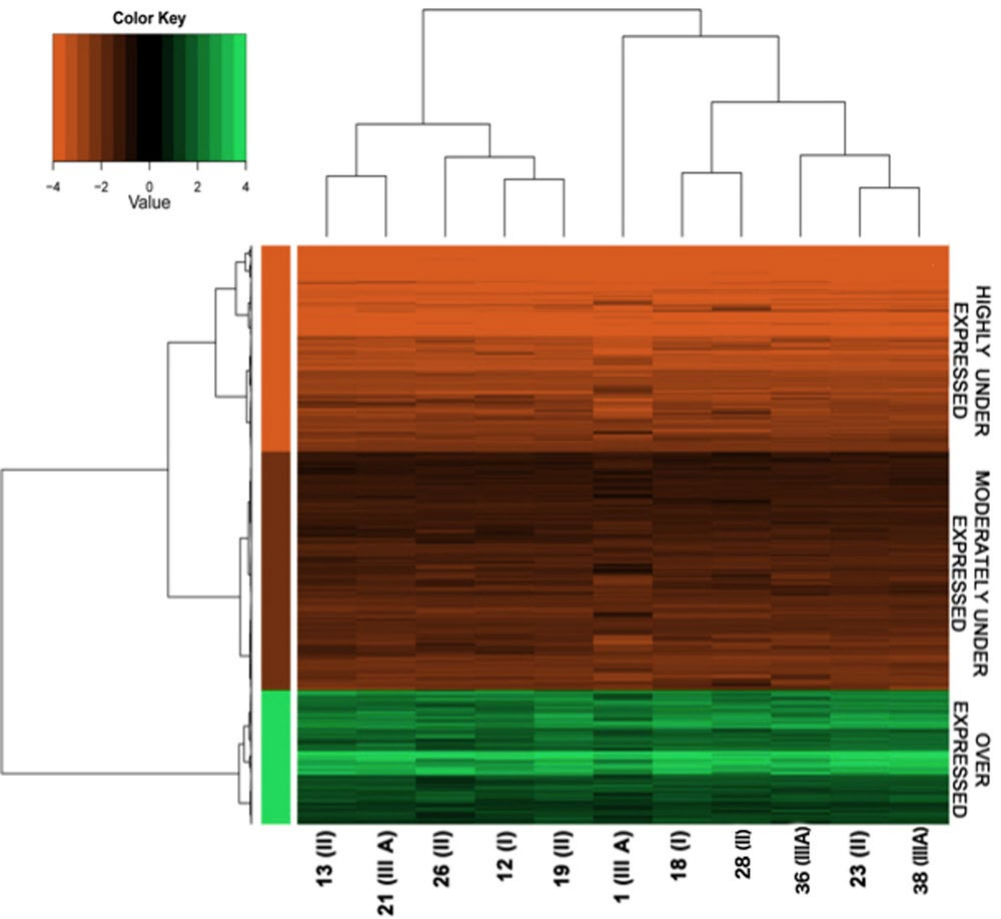
Heatmap of all the DE genes. Heatmap of 3,988 DE genes. DE genes were first filtered by removing all the genes that had an average expression level two times smaller or two times greater than control samples. After filtering, we obtained 3,988 DE genes that were used for cluster analysis by a Ward hierarchical clustering algorithm separately for samples and genes. To cluster samples, we used the matrix of the Pearson’s correlation coefficient; for genes, we used the matrix of the Euclidean distance. The cluster analysis and heat-map analysis were both performed using the R statistical environment. Clustering analysis revealed groups of genes and samples (reported on the bottom and expressing the tumour grade in brackets when available) with similar average expression levels, according to the colour key. Depending on the expression level, genes were segregated into 3 different clusters: over expressed genes (green, n = 925), moderately under expressed (dark red, n = 1642) and highly under expressed (brilliant red, n = 1421) compared with the normal lymph node.

The DNA microarray analysis was first focused on FL samples: the GEP was determined, and compared to that of normal lymph nodes from pooled healthy donors. In particular, a gene was assumed to be DE when the corrected *p*-value was lower than 0.01 and the fold change (log2 fold change) was ≥2. Applying such thresholds, 3988 DE genes, mostly under expressed, were detected and classified in 3 different clusters: over expressed genes (n = 925), moderately under expressed genes (n = 1642) and highly under expressed genes (n = 1421) (Fig. 1*;* raw data are deposited into the Gene Expression Omnibus (GEO) database (series entry GSE126247) (http://www.ncbi.nlm.nih.gov/geo/query/acc.cgi?acc=GSE126247).

No relevant difference in the stratification of the DE genes within the 11 FL samples emerged (see the upper dendrogram in Fig. 1), indicating a substantial homogeneity of the molecular characteristics of the samples, in agreement with the roughly homogenous clinical and pathological characteristic of our cohort. Microarray results were validated on those samples (nine) with enough residual RNA after the microarray analysis, performing RQ-PCR on some selected genes. The Pearson correlation coefficient values indicated a good correlation between the RQ-PCR and microarray expression data (Supplementary Table S3).

We next performed a functional annotation analysis (FAA) to identify the most altered biological processes. Particularly, looking at the Gene Ontology Biological Processes annotations (GOBP) we found that the DE genes are significantly associated to 474 groups (called “terms”) of potential functional distinction (Supplementary Dataset 1).

The functional annotation analysis (FAA) on the DE genes associated to the three clusters of the heatmap in Fig. 1, indicated that the biological processes associated to the over expressed DE genes were related to immune response, cell death, transport, chemotaxis and some peculiar signalling pathways such as TNF and NF-κB. Moderately under expressed genes were associated to biological processes related to cellular development, cell differentiation, cell motility and cytoskeleton organization, while the highly under expressed genes were associated with reproductive processes and cell cycle terms (Supplementary Datasets 2–4).

We then compared the gene expression profile of our FL cohort (henceforth addressed as the Florence Cohort) with other FL datasets, deposited into the GEO database (http://www.ncbi.nlm.nih.gov/geo/). Three different datasets (GSE32018, GSE9327 and GSE65135) that mostly matched our cohort’s characteristics were selected, and the microarray expression raw data were used to identify common differentially expressed genes. When comparing the profile of each dataset with that of the Florence Cohort, it emerged that the GSE65135 dataset had 641 DE genes, out of a total of 4151 DE genes, in common with the GEP of our cohort, hence displaying the higher similarity with the Florence Cohort (see the Venn diagram in Supplementary Fig. S1). The GSE65135 dataset was hence used for further comparisons.

### Analysis of DE genes associated with the transporter classification database (TCDB)

**Figure 2:**
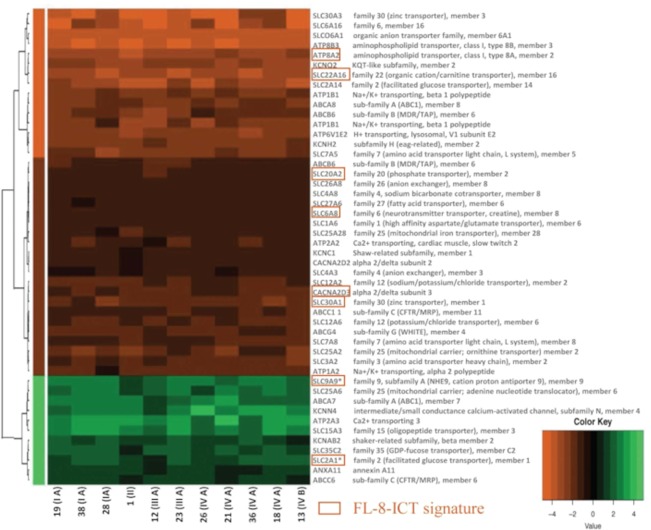
Heatmap of DE genes associated with transporters and ion channels. Heatmap of 48 DE genes associated with the TCDB, presented as an average of the expression level higher than 1 compared with the control. Samples were ordered in columns and genes in rows, gene names were reported on the right and samples on the bottom expressing the tumour grade in brackets when available. The samples were organized based on the FL stage. Expression levels are reported according to the colour key. Three different clusters were obtained: over expressed genes (green, n = 11), moderately under expressed genes (dark red, n = 22) and highly under expressed genes (brilliant red, n = 15). DE ICT genes shared with the GSE65135 dataset are highlighted in red boxes and named FL-8-ICT signature. Among them, *SLC2A1* and *SLC9A9*, marked with * are over expressed in both datasets.

We then performed a more focused analysis selecting, among the DE genes, the probes associated with ICT-encoding genes, according to the Transporter Classification Database (TCDB) (http://www.tcdb.org/hgnc_explore.php) as in^25^. Selecting those genes that presented an average expression level higher than 1 compared to the control, we identified 46 DE genes (Supplementary Table S4 and Supplementary Dataset 5) included in two main groups: those coding for transporters (n = 39; 85%) and those coding for ion channels (n = 7; 15%). Most of the DE genes encoded solute carriers (SLC, n = 25). Other categories included ATP-binding cassettes (ABC, n = 6), ATPases (n = 7), potassium channels (n = 5), calcium channels (n = 2), and one annexin (ANXA11). Seventy-six percent of these 46 DE ICT genes were under expressed, in line with the percentage of under expressed genes in the whole set of DE genes. Only 11 out of 46 genes were over expressed (Supplementary Table S4). An additional heatmap, restricted on the representation of ICT, was generated (Fig. 2). The profile confirms homogeneity among samples, as occurred for the whole GEP.

**Figure 3:**
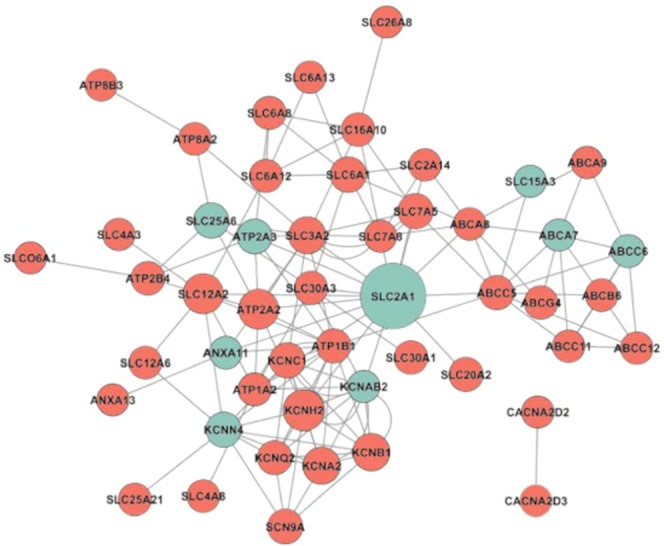
Ion channel and transporter gene network based on the literature data. Literature-based network of our DE ion channel and transporter genes performed using the PQ algorithm and generated using the Cytoscape tool. Gene connections are based on and filtered by expression/localization methods including: microscopy techniques, electrophoresis, western, Southern and northern blots, fluorometry, brain mapping, GE profiling, gene silencing, ligase chain reaction, PCR techniques, luminescent measurements, protein array analysis, *in situ* hybridization and histochemistry. Genes were represented by nodes (over expressed genes are shown in light green, and under expressed genes are shown in light red), connections (edges) represented the co-occurrence of at least two different genes in GEP papers. The node size is proportional to the number of occurrences related to the gene; SLC2A1 (GLUT-1) emerged as the hottest ion channel and transporter gene in the literature.

We then analysed whether and how the 46 DE ICT genes were related to each other. Using the PQ software, we found co-occurring ICT DE genes in either the same article abstracts or the image captions^26^. The final network, constituted by 50 nodes and 136 edges, was visualized through the Cytoscape platform (Fig. 3). Over- and under-expressed genes (nodes) were evidenced by a colour code (light-green and light-red coloured, respectively). A main network emerged, in which all the genes were found to be linked, except two (*CACNA2D2* and *CACNA2D3*, both under expressed) that were connected to each other but disconnected from the other ICT genes. *SLC2A1*, encoding the glucose transporter 1 (Glut1), represents the core of this connection network and is the most cited ICT in the literature, as indicated by the node size. Most of the solute carriers and potassium channels surrounded the edge of the network, and all of them were strictly connected. ATPases, on the other hand, were peripheral in the network and had fewer links with the other ICT functions and roles.

The 46 DE ICT genes identified in the Florence Cohort (FC-46-ICT signature) were then compared with the ICT DE genes of the GSE65135 dataset. 103 ICT genes were differentially expressed in the GSE65135 dataset (Supplementary Table S5). The overlap between the two datasets showed 8 commonly DE ICT genes (highlighted in red in *Fig.* *2*, and named FL-8-ICT signature), which hence potentially represent the ICT gene signature of Follicular Lymphoma. Among them, *SLC2A1* and *SLC9A9* (encoding the Na^+^/H^+^ antiporter NHE9) were present and over expressed in both datasets.

### Correlation of the ICT gene signature with clinical parameters

We then evaluated whether any relationship existed between the FL-DE-ICT genes and clinical data. Due to the small size of our cohort, it was impossible to perform survival analyses, using state-of-the-art methods for microarray experiments (rbsurv Package, 1, from Bioconductor in R). On the contrary, an association analysis with clinical parameters based on a generalized linear model was applicable, although limited to the Florence Cohort. In fact the clinical parameters relative to the datasets GSE32018, GSE9327 and GSE65135 were not available. No statistically significant association emerged between clinical parameters and DE-ICT genes. The extension to the whole set of DE genes led to identify 27 DE genes (involved in different processes such as cell differentiation, regulation of transcription and cytoskeleton organization) associated with the disease stage (Supplementary Table S6). Although these genes showed a significant nominal p-value, they did not pass the canonical threshold (p < 0.05) after the multiple test correction, required to correct the p-value when testing the statistically significance of a large group of genes.

We then analyzed the DE genes in relapsed vs non-relapsed FL patients. Once applied to the whole set of DE gene, this analysis evidenced 1580 differentially expressed genes between the two cohorts, with a p value of 0.05. When restricted to the ICT genes, it emerged that 38 DE ICT genes (16 over expressed and 22 under expressed) characterized relapsed patients (Table 2). All the ICT categories (ABC transporters, ATPases, K^+^ channels and solute carriers) were roughly equally distributed between over and under expressed genes, whereas only a sodium channel encoding gene (*SCN2A2*) was over expressed. Notably, *KCNN4* encoding for K_Ca_3.1 potassium channels was under expressed, at difference from what observed in the whole set of FL samples (over expressed in Fig. 2). Seven out of the 38 DE ICT genes overlapped with the FC-46-ICT signature and two (*SLC2A1* and *SLC6A8*, encoding for a creatine transporter) with the FL-8-ICT signature. At difference from what occurs in the whole cohort of FL patients, *SLC2A1* was highly under expressed in relapsed FL patients (see the highlighted genes in Table 2).

**Table 2:** DE ICT genes in relapsed versus not-relapsed FL patients: 16 genes were over expressed in relapsed patients (a) versus not relapsed and 22 under expressed (b).

gene function	gene name	Log2FC	p-value	gene type
**(a) over expressed genes**				
ATP-binding cassette	ABCC4	3,61	0,00692	subfamily C member 4
	ABCA2	3,03	0,01639	subfamily A member 2
ATP-ase	ATPAF2	5,52	0,00221	mitochondrial F1 complex assembly factor 2
	ATP5S	2,94	0,01776	H^+^ transporting, mitochondrial Fo complex subunit s
	ATP5E	2,58	0,03017	H^+^ transporting, mitochondrial F1 complex, epsilon subunit
Calcium Channel	CACNA1A	2.46	0,03783	P/Q type, alpha 1A subunit
Potassium Channel	KCNMB1	2,95	0,02260	subfamily M regulatory beta subunit 1
	KCNS1	2,83	0,02327	modifier subfamily S member 1
	**KCNQ2**	2,49	0,03558	KQT-like subfamily, member 2
Sodium Channel	SCN2A2	3,28	0,02613	voltage-gated channel alpha subunit 2
Solute Carrier	**SLC4A3**	3,11	0,02302	family 4 (anion exchanger), member 3
	SLC24A1	2,87	0,01869	family 24 (sodium/potassium/calcium exchanger), member 1
	SLC22A7	2,58	0,03126	family 22 (organic anion transporter), member 7
	SLC13A2	2,51	0,03331	family 13 (sodium-dependent dicarboxylate transporter), member 2
	SLC1A1	2,49	0,04442	family 1 (neuronal/epithelial high affinity glutamate transporter), member 1
	SLC23A3	2,48	0,04681	family 23 (nucleobase transporters), member 3
**(b) under expressed genes**				
ATP-binding cassette	**ABCB6**	−2,29	0, 04865	sub-family C (CFTR/MRP), member 6
	ABCA1	−2,48	0,03744	sub-family A, member 1
	ABCC5	−3,08	0,02647	sub-family C (CFTR/MRP), member 5
Annexin	ANXA3	−2,27	0,04918	annexin A3
ATP-ase	ATP9A	−2,72	0,02390	phospholipid transporting 9A
	ATP5H	−2,77	0,02222	H^+^ transporting, mitochondrial Fo complex subunit D
	ATP6V0D2	−2,98	0,01841	H^+^ transporting, lysosomal V0 subunit D2
	ATP2C1	−3,09	0,02373	Ca^2+^ transporting 1
Calcium Channel	CACNB4	−3,06	0,01363	beta 4 subunit
Potassium Channel	**KCNN4**	−2,33	0,04929	intermediate/small conductance calcium-activated channel, subfamily N, member 4
	KCNJ2	−2,96	0,01598	subfamily J member 2
	KCNIP2	−3,05	0,02790	interacting protein 2
	KCNE2	−4,23	0,00266	subfamily E regulatory subunit 2
Solute Carrier	SLC2A1	−2,33	0,04710	family 2 (facilitated glucose transporter), member 1
	SLC24A4	−2,39	0,04487	family 24 (sodium/potassium/calcium exchanger), member 4
	SLC24A3	−2,43	0,03885	family 24 (sodium/potassium/calcium exchanger), member 3
	SLC6A8	−2,47	0,04353	family 6 (neurotransmitter transporter, creatine), member 8
	SLC39A12	−2,63	0,04616	family 39 (zinc transporter), member 12
	SLC7A4	−2,72	0,02491	family 7 (amino acid transporter light chain, L system), member 4
	**SLC20A2**	−2,97	0,03109	family 20 (phosphate transporter), member 2
	SLCO2A1	−3,27	0,01049	organic anion transporter family member 2A1
	SLC26A4	−3,45	0,00937	family 26 (anion exchanger), member 4

ICT genes overlapping with FC-46-ICT signature are highlighted in **bold**.

ICT genes overlapping with FC-8-ICT signature are highlighted in **bold and underlined**.

All the overlapping genes showed the same trend in both cohorts.

### Comparison of ICT gene signature in FL and DLBCL samples

Since FL can progress either to a similar, although more aggressive, FL or to a DLBCL^3,4^, we also determined the ICT-GEP of DLBCL. To this purpose, we first analysed the two primary DLBCL samples present in our cohort (Table 1), whose RIN values allowed the accomplishment of whole genome analysis (Supplementary Table S1). Their ICT signature was determined, using the same analytical procedure used in FLs’ GE analysis. 26 DE ICT genes emerged, 19 of which overlapped with the FC-46-ICT signature (Table 3) and four (*SLC22A16*, *SLC30A1*, *SLC6A8* and *CACNA2D3*) with the FL-8-ICT signature. All the overlapping genes (underlined in Table 3) showed the same expression trend in both cohorts. The 19 overlapping genes encoded solute carriers (SLC, n = 10), ATP-binding cassettes (ABC, n = 3), ATPases (n = 3) and ion channels (n = 3). Eighty-nine percent (17/19) of these 19 genes were under expressed. The seven ICT DE genes not overlapping between the DLBCL and the FC-46-ICT signature (reported in bold in Table 3) were the followings: *ANXA8*, *ATP9A*, *CACNA1E*, *CACNA1I*, *SLC26A1*, *SLC27A1*, *SLC7A4*. This is apparently a DLBCL-specific ICT signature. Because of the limited number of DLBCL samples present in our cohort the DLBCL-ICT profile was validated with the ICT GEP obtained from a publicly available dataset deposited into the GEO database. We chose (on the basis of the characteristic of our cohort) the dataset GSE12195 that contains microarray data from 71 DLBCL, and compared the GEP with that of centrocytes purified from the tonsils of 5 healthy subjects from the same dataset^27^.

**Table 3:** DE ICT genes in DLBCL primary samples (two primary samples: 14 and 30). LOD, logarithm of odd calculated by Newton test. a: Over expressed genes ; b: under expressed genes. DE ICT genes (n = 19) in primary DLBCL overlapping with the FC-42-ICT signature are underlined, and all of them showed the same trend in both cohorts. The genes ATP2A3, CACNA1I, SLC27A1 and ATP9A, highlighted in **bold**, resulted deregulated in both our cohort and in the GSE12195 dataset (Table 4).

gene function	gene name	Log2FC DLBCL 14	Log2FC DLBCL 30	LOD	gene type
**(a) over expressed genes**					
ATP-ase	ATP2A3	3,30	3,91	3,53	Ca^2+^ transporting 3
Calcium Channel	CACNA1E	4,84	3,49	4,63	voltage-gated subunit alpha1 E
	**CACNA1I**	4,39	3,74	3,96	voltage-gated subunit alpha1 I
Solute Carrier	SLC26A1	3,80	3,77	3,87	family 26 (anion exchanger), member 1
	SLC15A3	2,92	3,16	2,27	family 15 (oligopeptide transporter), member 3
	**SLC27A1**	2,33	2,40	1,19	family 27 (fatty acid transporter), member 1
**(b) under expressed genes**					
ATP-binding Cassette	ABCA8	−3,66	−3,38	3,18	subfamily A, member 1
	ABCB6	−3,12	−2,89	2,22	sub-family B (MDR/TAP), member 6
	ABCC11	−2,73	−2,55	1,52	subfamily C (CFTR/MRP), member 11
Annexin	ANXA8	−2,47	−2,37	1,23	annexin A8
ATP-ase	ATP1A2	−3,10	−2,71	2,06	Na^+^/K^+^ transporting subunit alpha 2
	ATP8B3	−5,14	−5,57	7,30	aminophospholipid transporter, class I, type 8B, member 3
	**ATP9A**	−2,94	−2,57	1,58	phospholipid transporting 9A
Calcium Channel	CACNA2D3	−3,20	−2,73	2,21	alpha 2/delta subunit 3
Potassium Channel	KCNH2	−3,68	−2,93	2,84	subfamily H (eag-related), member 2
	KCNQ2	−4,46	−3,99	4,64	KQT-like subfamily, member 2
Solute Carrier	SLC12A6	−2,53	−2,25	1,21	family 12 (potassium/chloride transporter), member 6
	SLC22A16	-4,38	-4,23	4,93	family 22 (organic anion transporter), member 16
	SLC25A2	−3,06	−3,54	2,81	family 25 (mitochondrial carrier; ornithine transporter) member 2
	SLC2A14	−4,41	−2,76	3,02	family 2 (facilitated glucose transporter), member 14
	SLC30A1	−2,82	−2,35	1,57	family 30 (zinc transporter), member 1
	SLC30A3	−4,68	−3,45	4,26	family 30 (zinc transporter), member 3
	SLC6A16	−4,53	−5,04	5,96	family 6 (neurotransmitter transporter), member 16
	SLC6A8	−2,50	−2,39	1,19	family 6 (neurotransmitter transporter, creatine), member 8
	SLC7A4	−3,18	−3,34	2,77	family 7 (amino acid transporter light chain, L system), member 4
	SLCO6A1	−5,49	−5,33	7,22	organic anion transporter family, member 6A1

The expression analysis was performed as for the other datasets and 42 DE ICT genes emerged, 20 being under expressed, and 34 over expressed (Table 4). In the GSE12195 dataset the DE ICT genes are compared to centrocytes, i.e. “activated” B lymphocytes, while the data obtained from our patients’ cohort compares microarray data from patients to those of normal, resting B cells. Nevertheless, 4 out of the 26 DE ICT genes of our two primary DLBCL samples overlapped with the ICT profile in the GSE12195 dataset: *ATP2A3*, *ATP9A*, *CACNA1I* and *SLC27A1* (highlighted in **bold** in Tables 3 and 4). Among them, *ATP9A*, *CACNA1I* and *SLC27A1* matched the 7 DLBCL-DE ICT specific genes identified in our cohort (reported in bold and squared in Table 3), and may hence represent the true DLBCL-specific ICT signature.

**Table 4:** DE ICT genes in DLBCL primary samples from the GSE12195 dataset compared with a cohort of 5 healthy centrocytes present in the same dataset. The log2 fold change and the adjusted p-value (corrected with the FDR calculated according to the Benjamini & Hochberg method) are reported. Genes have been considered deregulated when showing a log2 Fold Change ≥2 and with an adjusted p-value < 0,01, and 42 DE ICT emerged. Transcripts from 12 genes are recognized by multiple probes, all presenting the same trend and similar FC values among each other.

Gene function	Gene name	Log2FC	adj.P.Val	Gene type
**(a) over expressed genes**				
ATP-binding Cassette	ABCC3	3,03	4,9E-04	ATP binding cassette subfamily C member 3
Annexin	ANXA1	4,98	1,9E-17	annexin A1
	ANXA2	2,87	1,2E-14	annexin A2
	ANXA2	2,84	4,2E-14	annexin A2
	ANXA2	2,83	2,8E-14	annexin A2
	ANXA2P3	2,22	6,3E-08	annexin A2 pseudogene 3
ATP-ase	**ATP9A**	3,15	4,8E-05	ATPase phospholipid transporting 9A (putative)
	ATP2B4	2,92	3,4E-06	ATPase plasma membrane Ca^2+^ transporting 4
	ATP11A	2,82	6,8E-05	ATPase phospholipid transporting 11A
	ATP1B1	2,57	7,0E-05	ATPase Na^+^/K^+^ transporting subunit beta 1
	ATP11A	2,37	6,8E-07	ATPase phospholipid transporting 11A
	ATP1B1	2,28	5,0E-04	ATPase Na^+^/K^+^ transporting subunit beta 1
	ATP11A	2,16	2,0E-07	ATPase phospholipid transporting 11A
Calcium Channel	CACNA2D1	3,58	5,8E-07	calcium voltage-gated channel auxiliary subunit alpha2delta 1
Potassium Channel	KCNJ10	4,45	9,7E-08	potassium voltage-gated channel subfamily J member 10
	KCNJ8	4,28	7,3E-11	potassium voltage-gated channel subfamily J member 8
	KCNMA1	2,83	3,7E-07	potassium calcium-activated channel subfamily M alpha 1
	KCNS3	2,16	2,4E-03	potassium voltage-gated channel modifier subfamily S member 3
	KCNJ5	2,02	1,3E-03	potassium voltage-gated channel subfamily J member 5
Solute Carrier	SLC1A3	5,05	5,1E-15	solute carrier family 1 member 3
	SLCO2B1	4,21	2,8E-09	solute carrier organic anion transporter family member 2B1
	SLC40A1	4,04	1,9E-03	solute carrier family 40 member 1
	SLC31A2	3,96	4,9E-11	solute carrier family 31 member 2
	SLC2A10	3,83	6,5E-06	solute carrier family 2 member 10
	SLC1A3	3,65	1,7E-04	solute carrier family 1 member 3
	SLC22A3	3,61	7,6E-03	solute carrier family 22 member 3
	SLCO2B1	2,80	4,8E-09	solute carrier organic anion transporter family member 2B1
	SLC26A11	2,66	1,4E-08	solute carrier family 26 member 11
	SLC37A3	2,59	9,0E-07	solute carrier family 37 member 3
	SLC8A1	2,45	4,4E-04	solute carrier family 8 member A1
	SLC27A5	2,36	2,7E-04	solute carrier family 27 member 5
	SLCO2A1	2,33	1,4E-06	solute carrier organic anion transporter family member 2A1
	**SLC27A1**	2,17	1,2E-04	solute carrier family 27 member 1
	SLC7A7	2,14	2,2E-06	solute carrier family 7 member 7
**(b) under expressed genes**				
ATP-ase	ATP2B2	−2,19	4,2E-03	ATPase plasma membrane Ca^2+^ transporting 2
	ATP6V1G3	−2,33	4,9E-05	ATPase H+ transporting V1 subunit G3
	ATP2A3	−2,50	8,1E-04	ATPase sarcoplasmic/endoplasmic reticulum Ca^2+^ transporting 3
	ATP2A3	−2,60	7,9E-04	ATPase sarcoplasmic/endoplasmic reticulum Ca^2+^ transporting 3
Calcium Channel	**CACNA1I**	−2,10	1,6E-03	calcium voltage-gated channel subunit alpha1 I
Potassium Channel	KCNN3	−2,05	7,6E-03	potassium calcium-activated channel subfamily N member 3
	KCNA6	−2,19	7,1E-03	potassium voltage-gated channel subfamily A member 6
	KCNT1	−2,28	2,1E-03	potassium sodium-activated channel subfamily T member 1
	KCNK12	−3,14	1,6E-03	potassium two pore domain channel subfamily K member 12
Solute Carrier	SLC29A2	−2,02	7,4E-03	solute carrier family 29 member 2
	SLC24A1	−2,05	4,6E-03	solute carrier family 24 member 1
	SLC4A8	−2,05	6,4E-03	solute carrier family 4 member 8
	SLC16A11	−2,16	2,3E-03	solute carrier family 16 member 11
	SLC16A8	−2,17	1,6E-04	solute carrier family 16 member 8
	SLC34A1	−2,18	7,0E-03	solute carrier family 34 member 1
	SLC15A2	−2,20	4,3E-03	solute carrier family 15 member 2
	SLC4A8	−2,23	5,5E-05	solute carrier family 4 member 8
	SLC30A4	−2,55	9,1E-04	solute carrier family 30 member 4
	SLC30A4	−2,81	3,6E-03	solute carrier family 30 member 4
	SLC30A4	−3,15	2,7E-03	solute carrier family 30 member 4

DE ICT genes overlapping with the FC-42-ICT signature are underlined (ATP1B1 and ATP2A3) and both of them showed the same trend in both cohorts. All the other genes are not overlapping between the DLBCL and the FL ICT and apparently belong to an ICT signature specific of DLBCL.

The genes ATP2A3, CACNA1I, SLC27A1 and ATP9A, highlighted in **bold**, resulted deregulated in both the GSE12195 dataset and in our DLBCL cohort (Table 3).

## Discussion

In the present study we determined the expression profile of ICT genes (ICT-GEP) of Follicular Lymphoma (FL) and of Diffuse Large B Cell Lymphoma (DLBCL), with the aim to identifying different profiles related to disease progression and therefore with their potential translational relevance. cDNA microarray data were collected both from samples of a patients’ cohort enrolled for this study (the Florence cohort, FC), and from public datasets that matched the clinical and technical characteristics of our cohort. For the first time, it is here provided the ICT signature of FL, its variations during disease relapse and the different ICT profile of DLBCL.

The GEP of 11 FL consecutive samples from the FC was determined, from which 46 DE ICT genes emerged in the Florence Cohort and 8 of them were in common between the FC and the GSE65135 dataset (Fig. 2). The ICT profile of FLs showed an upregulation of K^+^ channels, witnessed by the over expression of *KCNN4* and *KCNAB2*, which encode for the alpha and beta subunits of Ca^2+^-dependent K^+^ channels, respectively. Notably, the Ca^2+^-dependent K^+^ channel K_Ca_3.1 encoded by *KCNN4*, is one of the two K^+^ currents (the other being K_v_1.3) involved in lymphocyte activation and proliferation, and its expression marks the differentiation step characterizing activated naïve B cells and IgD^+^ CD27^+^ memory B cells^19^. Thus *KCNN4* over expression supports the notion that B lymphocytes present in FLs are neoplastic germinal centre cells in an activated state. Other potassium channel encoding gene, such as *KCNA2* and *KCNH2* (encoding K_V_1.2 and Kv11.1, respectively) were under expressed, at difference from what occurs in other human cancers including leukemias^28^. Calcium channels encoding genes, in particular *CACNA2D2* and *CACNA2D3*, were also under expressed, suggesting that Ca^2+^ signalling is sustained by other Ca^2+^ transport mechanisms, possibly including ATPases, as witnessed by the over expression of *ATP2A3*. The over expression of two solute carriers, *SLC2A1* (encoding the glucose transporter member 1 (Glut-1)) and *SLC9A9* (encoding the Na^+^/H^+^ antiporter NHE9), merits attention. While NHE proteins contribute to determine the inverted pH characteristics of cancer cells extruding the protons deriving from (anaerobic) metabolism^29^, Glut-1 is the hallmark of the main metabolic pathway of cancers, i.e. aerobic glycolysis (the so-called Warburg effect). In lymphomas, Glut-1 expression is apparently involved in FL transformation^30^ and it is related to the NF-κB pathway, which turned out to be upregulated in FL from our FAA. Also the TNF pathway results upregulated in FL, and both of them are associated with the control of the metabolism^31,32^.

Notably, *SLC2A1* was also found at the main edge of the network involving all the DE ICT genes (Fig. 3), meaning that the other solute carriers and potassium channels surrounding the edge of the network are strictly connected with it. Overall, the ICT profile emerging from our data indicates that the main metabolic pathway of FL is represented by glycolysis, and that neoplastic B cells are “excitable”, although at a lesser extent compared to activated normal B lymphocytes. Indeed, in neoplastic B cells, the only relevant K^+^ channel is K_Ca_3.1, which could contribute to sustain cell proliferation^15,20^.

**Figure 4:**
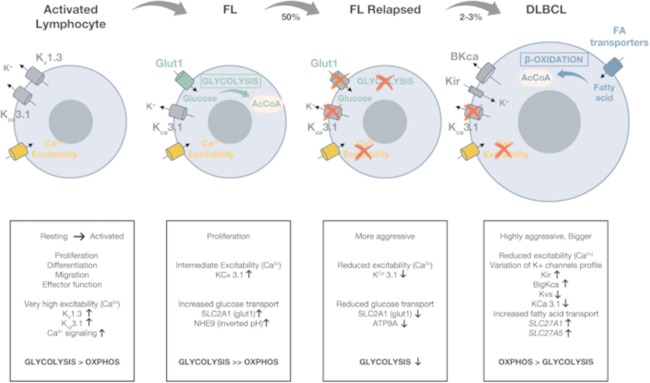
Overview of the ICT profile along lymphoma progression. Summary figure indicating the changes in the expression of the different ICT and the associated metabolic pathways along lymphoma progression. The represented ICT are described in detail in the text.

Conversely, both *SLC2A1* and *KCNN4* turned out to be under expressed in relapsed FL cases (Table 2). This suggests that, upon relapse, neoplastic B cells become even less activated and undergo a metabolic shift, leading to down regulation of glycolysis (Fig. 4). FL often acquire chemoresistance after relapse, and fatty acid metabolism has been linked with resistance to chemotherapeutics in multiple cancer types^33^. The glycolytic to oxidative shift is also confirmed by the overexpression (log2 fold change value 5.52) of *ATPAF2*, one of the factors involved in mitochondrial functioning^34^, and by the under expression of *ATP9A* (Table 2). A depletion of *ATP9A* has been found to cause retaining of Glut-1 in endosomes, probably by inhibiting its recycling, and hence reduces its expression on the cell surface^35^. *ATP9A* was also DE in DLBC patients, although with different directions in the Florence Cohort and in the GSE12195 dataset, respectively (Tables 3 and 4). Such discrepancy could be traced back to the fact that DE genes are compared to different controls (normal lymphocytes and centrocytes, respectively) in the two datasets. Nevertheless, the common dysregulation of this gene in both DLBCL and in the relapsed FL subgroup suggests its possible involvement in the progression of FL to the more aggressive DLBCL disease.

The changes in excitability and the shift from a glycolytic to an oxidative metabolic profile observed in relapsed FL is even more evident when analysing the DE-ICT profile of DLBCL (Tables 3, 4, Fig. 4). In DLBCL, in fact, both *CACNA1I* (and the correlated *CACNA1E*) which encodes the alpha subunit of the low voltage-activated, T-type calcium channel, and the *SLC27A1* solute carrier, fatty acid transporter, implicated in the uptake of fatty acids for further beta oxidation^36^, turned out to be DE. The dysregulation of Ca^2+^ channels encoding genes is also accompanied by a drastic shift of the profile of K^+^ channels. In fact, the two K^+^ channels which mark normal B lymphocytes (K_V_1.3 and K_Ca_3.1) are substituted by the over expression of genes encoding inward rectifier K^+^ channels (Kir) and Big K_Ca_ channels (BK) (*KCNJ8*, *KCNJ10*, *KCNJ5*, *KCNMA1* (Table 4 and Fig. 4).

*SLC27A1* deregulation (i.e. over expression in both the Florence cohort and the GSE12195 dataset) merits attention. *SLC27A1* has been associated with tumorigenesis^37^, since its increased levels would properly supply fatty acids from the surrounding adipocytes for β-oxidation, in turn providing an alternative pathway for acetyl CoA to be metabolized in the TCA cycle when cancer cells switch from a glycolytic to an oxidative metabolism^37^. Thus, the downregulation of Glut-1 and the upregulation of the fatty acid transporter *SLC27A1* in DLBCL indicate a shift from a glycolytic to an oxidative metabolism during lymphoma progression (Fig. 4). In agreement, oxidative enzymes (*ACAD10*, *ACAD8*, *ECHS1*, *HADHB*) turned out to be over expressed (log2FC > 0) in DLBCL (Supplementary Table S7). Consistently, the nuclear corepressor-encoding gene *NCoR1*, whose down-regulation has been reported to drive the switch towards oxidative metabolism^38^, results under expressed in DLBCL (Supplementary Table S8). In agreement with our data, an OxPhos subset of DLBCL, identified by a lower expression of *NCoR1*, has been recently described in^39,40^. Patients belonging to this subset might benefit from treatments perturbing the fatty acid oxidation program^39^. Although further functional studies must be conducted to confirm the therapeutic potential of the fatty acid oxidative pathway in aggressive lymphomas, *SLC27A1*, and the OxPhos pathway, might constitute a promising therapeutic target.

On the whole, starting from a wide transcriptomic analysis, our data indicate the occurrence of a progressive decrease in excitability and a metabolic shift during neoplastic progression of FL to more aggressive diseases. In particular, for the first time, we have characterized the ICT genes deregulated in relapsed FL patients, showing a decrease in excitability and glycolysis, which precedes the already described change towards an oxidative metabolism in DLBCL.

This finding might have a translational relevance through the identification of novel ICT related therapeutic targets that might overcome the chemoresistance that characterizes relapsed FL.

## Methods

### Patients

Lymph node surgical specimens were collected from patients at Careggi University Hospital (AOUC), Firenze (n = 50) and from San Jacopo Hospital, Pistoia (n = 4) during 3 years after informed written consent. The study was approved by the Ethical Committee of the AUOC and all research was performed in accordance with relevant guidelines/regulations. FL diagnosis was made according to 2008 World Health Organization Classification of Tumours of Haematopoietic and Lymphoid Tissues^41^. FL grading was assessed according to the REAL and WHO 3rd edition classifications according to the number of centroblasts (0–5; 6–15, >15 per high power field, respectively). Staging was assessed with the Ann Arbor classification system^42^.

### Primary samples

Part of each surgical specimen was cut into two corresponding pieces, one was processed for histological analysis and diagnosis, the other for total RNA extraction (Trizol, Invitrogen). 11 FL and 2 DLBCL samples displaying a RIN (RNA integrity number) >6 (Supplementary Table S1) at the Agilent 2100 Bioanalyzer (Agilent Technologies) were further processed for gene expression cDNA microarray analyses, and the GEP was compared with that of two available human lymph nodes (male and female donors, Maximum Value and Purity, MVP, Total RNA, Human Lymph Node, Stratagene) pooled and used as control reference.

### Real-time quantitative PCR (rqPCR)

Total RNA extraction, reverse transcription (RT) and RQ-PCR were performed as in^43^. Primers and detailed experimental procedure are reported online in Supplementary Table S3.

### Microarray data analyses

Purified RNA’s samples were prepared and compared as previously described in^25^. Each spot was first corrected for background intensities using the “normexp” approach of the background correct function. The between-array normalization and the quantile approach were performed according to the limma package’s functions. To choose the DE genes, we considered a threshold of 0.01 on the corrected p-value, plus an average cut-off of twofold changes. To find groups of co-regulated genes, we applied a hierarchical clustering algorithm (Ward method) to genes using the Euclidean distance as a measure of similarity.

All the genes analysed were annotated for their role in biological processes using the GO package of Bioconductor, (Gene Ontology consortium). Due to the small number of genes in some categories, two-sided Fisher’s exact test was applied.

DLBCL data analysis, due to the small samples size, was performed using the single slide method developed previously^44^ and implemented in the sma package (https://cran.r-project.org/package=sma) in R. Statistically significant differential expression was defined as an LOD score larger than 1.

#### Enrichment and functional annotation analysis (FAA) of the FL samples cohort

FAA of DE genes was based on Gene Ontology (GO) and (Kyoto Encyclopedia of Genes and Genomes (KEGG) terms enrichment, using the GOStats R package. For each GO term, a p-value is calculated representing the probability that the observed numbers of genes could have resulted from randomly distributing this GO term between the tested and reference groups.

We first found the set of all unique GO terms within the ontology that are associated with one or more of the genes of interest. Next, we determined how many of the selected DE genes are annotated at each term and how many of the genes represented on the microarray are annotated at the term. The test evaluated if there are more interesting genes at the term than one might expect by chance. For each GO term, a p-value is calculated representing the probability that the observed numbers of genes could have resulted from randomly distributing this GO term between the tested group and the reference group.

#### Analysis of microarray datasets

DE-ICT genes were identified as previously described.

GSE32018 contains data from 27 FLs compared to 7 lymph nodes. GSE9327 contains data from 33 FL, compared with 8 reactive lymph nodes. The GSE65135 contains data from 14 FLs, compared to the RNA from B cells from the tonsils of 5 healthy subjects.

The GSE12195 dataset, chosen on the basis of the characteristic of our cohort, contains data from 71 DLBCL and their DE genes are compared to centrocytes purified from the tonsils of 5 healthy subjects.

### TCGA meta-analysis

Was performed using the Cbioportal website^45,46^.

### Literature-based molecular network generation

Was performed using ProteinQuest (PQ, BioDigitalValley s.r.l.; www.proteinquest.com/pq/), a web-based platform for biomedical literature retrieval and analysis that retrieves PubMed abstracts and the text of the image captions from free full-text articles^26^.

## Supplementary information

Supplementary Information

Supplementary Dataset 1

Supplementary Dataset 2

Supplementary Dataset 3

Supplementary Dataset 4

Supplementary Dataset 5
