# Supplementary material for: The ion channels and transporters gene expression profile indicates a shift in excitability and metabolisms during malignant progression of Follicular Lymphoma
Source: Sci Rep. 2019 Jun 13;9:8586. doi: 10.1038/s41598-019-44661-x (PMC6565741; doi:10.1038/s41598-019-44661-x)
Supplement: Supplementary file 1 — Supplementary Information [file 41598_2019_44661_MOESM1_ESM.pdf]

Supplementary Materials for

The ion channels and transporters gene expression profile indicates a shift in excitability and metabolisms during malignant progression of Follicular Lymphoma.

Alberto Magi[+], Marika Masselli[+], Cesare Sala[+], Angela Guerriero, Pasquale Laise, Benedetta Puccini, Luigi Rigacci, Carla Breschi, Olivia Crociani, Serena Pillozzi and Annarosa Arcangeli *

+ These authors contributed equally to this work

*Corresponding author: Arcangeli, A. MD, PhD

This PDF file includes:

Figure S1. Cohort Comparison.

Table S1. RNA integrity number (RIN) values of Florence cohort's samples.

Table S2. Microarray expression data of Bcl2 and Bcl6 in the Florence cohort's samples.

Table S3. Validation of microarray results by rqPCR.

Table S4. DE genes associated to the Transporter Classification Database.

Table S5. DE genes associated with disease stage in the Florence Cohort.

Table S6. DE genes associated with disease stage.

Table S7. DE-ICT genes involved in the β Oxidation process in DLBCL primary samples from the GSE12195 dataset.

Table S8: Expression of the nuclear receptor corepressor 1 in DLBCL primary samples from the GSE12195 dataset.

Figure S1: Cohort Comparison

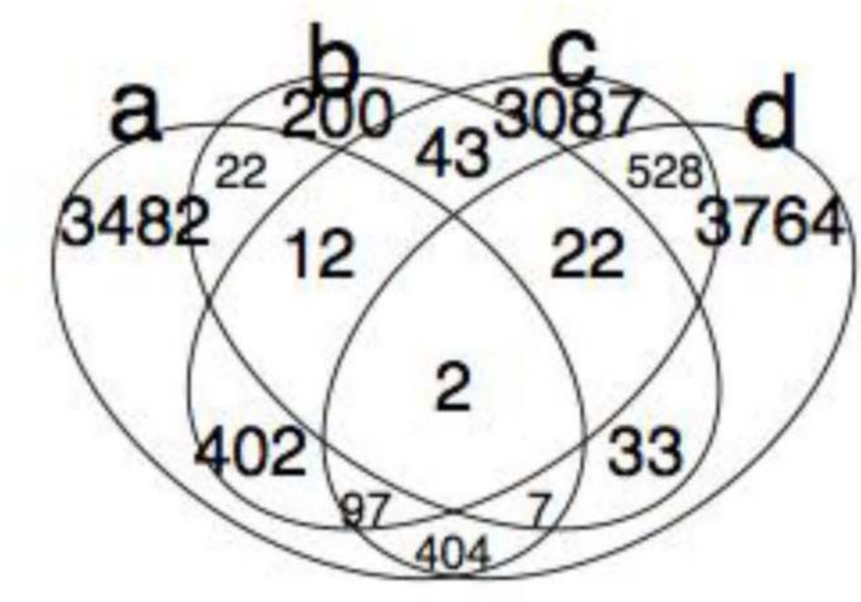

Figure S1: Number of differentially expressed genes in GSE21018 (a), GSE9327 (b), GSE65135 (c) and Florence Cohort (d). Intersection areas defines common genes amount.

Table S1: RNA integrity number (RIN) values of Florence cohort's samples

Sample number	Diagnosis	RNA integrity number (RIN)
1	FL III° A	6.7
2	DLBCL	**2.9**
7	FL I°	**8.5**
12	FL I°	8.4
13	FL II°	9.3
14	DLBCL	9.0
17	DLBCL	**2.7**
18	FL I°	9.3
19	FL II°	7.9
21	FL III° A	7.2
22	FL I°	**3.5**
23	FL II°	8.0
26	FL II°	6.0
28	FL II°	8.0
29	DLBCL	**2.5**
30	DLBCL	8.1
36	FL III°A	9.0
38	FL III°A	7.8

Table S1: RNA integrity number (RIN) values of samples of our cohort are reported. The RIN values were determined with Agilent Bioanalyzer 2100 and values lower than 6.0 are considered not sufficient to be used for microarray. Only samples that met both quality and quantity (>200 ng) of RNA criteria were processed for microarray analysis. Samples reported in bold did not accomplish both criteria reported above.

Table S2: Microarray expression data of Bcl2 and Bcl6 in the Florence cohort's samples

**Sample n.**	**Bcl6 (Log2FC)**	**Bcl2 (Log2FC)**	**Bcl2 (Log2FC)**
**Probe ID**		A_23_P208132	A_23_P352266
1	1,82003	3,64113	-0,31193
12	2,09432	0,25527	-1,18256
13	3,14358	0,13097	1,02327
18	1,64602	-1,45575	-0,97742
19	1,45178	-0,47214	-0,03115
21	1,55259	0,37275	-0,68883
23	0,15398	0,16939	0,43657
26	2,67157	-0,88528	-0,74257
28	0,37462	-0,27465	0,36434
36	3,46710	0,14142	-2,46415
38	1,63723	0,08046	0,43211

Table S2: Expression (Log2FC) of Bcl2 (2 different probes) and Bcl6 in the different samples of our cohort compared to a pool of healthy lymphnodes.

Table S3: Validation of microarray results by rqPCT

Samples	*KCNH2*				*CCND3*				*PGK1*				*SLC2A1*			
	rqPCR		***array***		***rqPCR***		***array***		***rqPCR***		***array***		***rqPCR***		***array***	
Control	1,0E+00		9,7E+00		1,0E+00		8,5E+00		1,0E+00		1,2E+01		1,0E+00		8,6E+00	
1	1,5E-03	dwn	6,8E+00	dwn	1,8E+01	up	9,8E+00	up	7,8E+00	up	1,3E+01	up	1,6E+00	up	4,2E-01	dwn
12	4,5E-03	dwn	7,4E+00	dwn	1,6E+02	up	1,1E+01	up	1,4E+00	up	1,4E+01	up	1,1E+00	up	9,6E+00	up
13	4,6E-01	dwn	7,7E+00	dwn	1,6E+02	up	1,1E+01	up	1,3E+02	up	1,4E+01	up	2,4E+02	up	1,1E+01	up
18	6,9E-04	dwn	7,0E+00	dwn	8,8E+01	up	1,1E+01	up	4,2E-01	dwn	1,4E+01	up	3,5E+00	up	1,0E+01	up
19	8,7E-03	dwn	7,2E+00	dwn	1,3E+01	up	1,1E+01	up	1,4E+00	up	1,3E+01	up	2,4E+00	up	1,0E+01	up
21	3,2E-03	dwn	6,9E+00	dwn	6,7E+00	up	1,1E+01	up	3,8E-01	dwn	1,3E+01	up	9,9E-01	dwn	1,0E+01	up
26	3,8E-02	dwn	7,3E+00	dwn	2,2E+01	up	1,0E+01	up	1,8E+01	up	1,3E+01	up	3,1E+00	up	1,0E+01	up
36	9,6E-04	dwn	7,0E+00	dwn	7,1E+02	up	1,1E+01	up	4,3E-01	dwn	1,4E+01	up	4,4E+00	up	1,1E+01	up
38	7,5E-01	dwn	6,9E+00	dwn	2,8E+04	up	1,1E+01	up	3,6E+01	up	1,4E+01	up	5,1E+00	up	1,0E+01	up

Table S3: Validation of microarray results of selected genes *KCNH2*, *CCND3*, *PGK* and *SLC21* (encoding for hERG1, Cyclin D3, PGK1 and Glut-1 respectively) by rqPCR using the primer pairs reported in Materials and Methods. Raw data of gene expression are reported and genes up-regulated compared to the respective control are indicated as up, genes down-regulated compared to the respective control are indicated as dwn.

Pearson correlation coefficient values were calculated for *KCNH2* (0,606), *CCND3* (0,725), *PGK1* (0,27) and *SLC2A1* (0,386) indicating a direct correlation between the rqPCR and microarray expression analysis.

Table S4: DE genes associated to the Transporter Classification Database

gene function	gene name	Log2FC	P-value	gene type
**ATP-binding cassette**	*ABCA7*	2,33	0,00075	sub-family A (ABC1), member 7
	ABCC6	1,12	0,00399	sub-family C (CFTR/MRP), member 6
**Annexin**	*ANXA11*	1,16	0,00315	annexin A11
**ATP-ase**	*ATP2A3*	2,99	0,00066	Ca^{2+} transporting 3
**Potassium Channel**	*KCNN4*	2,69	0,00312	intermediate/small conductance calcium-activated channel, subfamily N, member 4
**Solute Carrier**	*SLC15A3*	2,66	0,00244	family 15 (oligopeptide transporter), member 3
	SLC9A9*	2,38	0,00043	family 9, subfamily A (NHE9, cation proton antiporter 9), member 9
	SLC25A6	2,05	0,00079	family 25 (mitochondrial carrier; adenine nucleotide translocator), member 6
	SLC2A1*	1,66	0,00105	family 2 (facilitated glucose transporter), member 1
	SLC35C2	1,65	0,00745	family 35 (GDP-fucose transporter), member C2
	KCNAB2	1,51	0,00112	shaker-related subfamily, beta member 2

gene function	gene name	Log2FC	P-value	gene type
ATP-binding cassette	*ABCC11*	*-1,61*	*1,58E-11*	*sub-family C (CFTR/MRP), member 11*
	ABCG4	*-1,72*	*6,15E-09*	*sub-family G (WHITE), member 4*
	ABCB6	*-1,86*	*0,00032*	*sub-family B (MDR/TAP), member 6*
	ABCA8	*-2,28*	*1,58E-07*	*sub-family A (ABC1), member 8*
ATP-ase	*ATP8B3*	*-4,25*	*1,90E-09*	*aminophospholipid transporter, class I, type 8B, member 3*
	ATP2A2	*-1,4*	*0,00175*	*Ca^{2+} transporting, cardiac muscle, slow twitch 2*
	ATP1A2	*-1,95*	*0,00027*	*Na^{+}/K^{+} transporting, alpha 2 polypeptide*
	ATP1B1	*-2,47*	*2,21E-05*	*Na^{+}/K^{+} transporting, beta 1 polypeptide*
	ATP6V1E2	*-2,77*	*6,40E-06*	*H^{+} transporting, lysosomal, V1 subunit E2*
	ATP8A2	*-3,21*	*1,40E-07*	*aminophospholipid transporter, class I, type 8A, member 2*
Calcium Channel	***CACNA2D3***	*-1,79*	*2,04E-05*	*alpha 2/delta subunit 3*
	CACNA2D2	*-1,14*	*3,69E-05*	*alpha 2/delta subunit 2*
Potassium Channel	*KCNQ2*	*-3,04*	*2,32E-08*	*KQT-like subfamily, member 2*
	KCNH2	*-2,55*	*1,30E-06*	*subfamily H (eag-related), member 2*
	KCNC1	*-1,08*	*3,16E-08*	*Shaw-related subfamily, member 1*
Solute Carrier	*SLC4A3*	*-1,04*	*0,0037*	*family 4 (anion exchanger), member 3*
	SLC25A28	*-1,26*	*0,00488*	*family 25 (mitochondrial iron transporter), member 28*
	SLC26A8	*-1,34*	*1,66E-07*	*family 26 (anion exchanger), member 8*
	SLC1A6	*-1,39*	*3,79E-10*	*family 1 (high affinity aspartate/glutamate transporter), member 6*
	SLC20A2	*-1,39*	*0,00039*	*family 20 (phosphate transporter), member 2*
	SLC27A6	*-1,4*	*1,71E-06*	*family 27 (fatty acid transporter), member 6*
	SLC6A8	*-1,4*	*8,75E-09*	*family 6 (neurotransmitter transporter, creatine), member 8*
	SLC4A8	*-1,4*	*9,70E-07*	*family 4, sodium bicarbonate cotransporter, member 8*
	SLC7A8	*-1,54*	*4,14E-05*	*family 7 (amino acid transporter light chain, L system), member 8*
	SLC12A6	*-1,65*	*1,50E-08*	*family 12 (potassium/chloride transporter), member 6*
	SLC3A2	*-1,8*	*7,25E-05*	*family 3 (amino acid transporter heavy chain), member 2*
	SLC12A2	*-1,84*	*3,67E-05*	*family 12 (sodium/potassium/chloride transporter), member 2*
	SLC30A1	*-1,94*	*5,60E-06*	*family 30 (zinc transporter), member 1*
	SLC25A2	*-1,96*	*9,33E-05*	*family 25 (mitochondrial carrier; ornithine transporter) member 2*
	SLC7A5	*-2,4*	*0,00018*	*family 7 (amino acid transporter light chain, L system), member 5*
	SLC2A14	*-3,4*	*3,15E-05*	*family 2 (facilitated glucose transporter), member 14*
	SLC22A16	*-3,52*	*1,19E-09*	*family 22 (organic cation/carnitine transporter), member 16*
	SLC6A16	*-3,71*	*0,00199*	*family 6, member 16*
	SLC30A3	*-3,98*	*0,0006*	*family 30 (zinc transporter), member 3*
	SLCO6A1	*-4,1*	*1,52E-12*	*organic anion transporter family, member 6A1*

Table S4: DE genes associated to the Transporter Classification Database (TCDB). In the upper table are reported over expressed genes, in the lower table under expressed genes. The 8 Ion Channel and Transporters (ICT) DE genes present in both the Florence Cohort and the GSE65135 dataset are underlined. Genes marked with * present the same trend (over or down expressed) in either datasets.

Transcripts from ABCB6 and ATP1B1 genes are detected by 2 different probes on the array. Both readings present the same trend, and in the table is reported the average log2FC value.

Reported genes presented an average of the expression level higher than 1 compared with the control, with *p*-value (adjusted according to Bonferroni's method) < 0.01.

Table S5: DE ICT genes in the GSE65135 dataset

Gene Function	Gene Type	Gene Name
Annexin	annexin A1	ANXA1
	annexin A2	ANXA2
	annexin A2 pseudogene 2	ANXA2P2
	annexin A3	ANXA3
ATP-ase	ATP synthase mitochondrial F1 complex assembly factor 1	ATPAF1
	ATPase, aminophospholipid transporter, class I, type 8A, member 2	ATP8A2
	ATPase, aminophospholipid transporter, class I, type 8B, member 1	ATP8B1
	ATPase, aminophospholipid transporter, class I, type 8B, member 2	ATP8B2
	ATPase, Ca++ transporting, plasma membrane 1	ATP2B1
	ATPase, Ca++ transporting, plasma membrane 4	ATP2B4
	ATPase, Ca++ transporting, type 2C, member 1	ATP2C1
	ATPase, class II, type 9A	ATP9A
	ATPase, class V, type 10A	ATP10A
	ATPase, H+ transporting, lysosomal 16kDa, V0 subunit c	ATP6V0C
	ATPase, H+ transporting, lysosomal 21kDa, V0 subunit b	ATP6V0B
	ATPase, H+ transporting, lysosomal 34kDa, V1 subunit D	ATP6V1D
	ATPase, H+ transporting, lysosomal 56/58kDa, V1 subunit B2	ATP6V1B2
	ATPase, H+ transporting, lysosomal accessory protein 1	ATP6AP1
	ATPase, Na+/K+ transporting, alpha 1 polypeptide	ATP1A1
	ATPase, Na+/K+ transporting, alpha 4 polypeptide	ATP1A4
	ATPase, Na+/K+ transporting, beta 4 polypeptide	ATP1B4
ATP-binding	ATP-binding cassette, sub-family A (ABC1), member 1	ABCA1
	ATP-binding cassette, sub-family A (ABC1), member 9	ABCA9
	ATP-binding cassette, sub-family C (CFTR/MRP), member 3	ABCC3
	ATP-binding cassette, sub-family C (CFTR/MRP), member 4	ABCC4
	ATP-binding cassette, sub-family C (CFTR/MRP), member 9	ABCC9
	ATP-binding cassette, sub-family D (ALD), member 2	ABCD2
	ATP-binding cassette, sub-family F (GCN20), member 3	ABCF3
	ATP-binding cassette, sub-family G (WHITE), member 2 (Junior blood group)	ABCG2
Calcium Channel	calcium channel, voltage-dependent, alpha 2/delta subunit 1	CACNA2D1
	calcium channel, voltage-dependent, alpha 2/delta subunit 3	CACNA2D3
	calcium channel, voltage-dependent, beta 2 subunit	CACNB2
	calcium channel, voltage-dependent, beta 3 subunit	CACNB3

	calcium channel, voltage-dependent, T type, alpha 1G subunit	CACNA1G
Potassium Channel	Kv channel interacting protein 2	KCNIP2
	potassium channel, calcium activated large conductance subfamily M alpha, member 1	KCNMA1
	potassium channel, inwardly rectifying subfamily J, member 10	KCNJ10
	potassium channel, inwardly rectifying subfamily J, member 2	KCNJ2
	potassium channel, inwardly rectifying subfamily J, member 5	KCNJ5
	potassium channel, inwardly rectifying subfamily J, member 8	KCNJ8
	potassium channel, sodium activated subfamily T, member 2	KCNT2
	potassium channel, two pore domain subfamily K, member 1	KCNK1
	potassium channel, two pore domain subfamily K, member 10	KCNK10
	potassium channel, two pore domain subfamily K, member 5	KCNK5
	potassium channel, voltage gated shaker related subfamily A, member 2	KCNA2
	potassium channel, voltage gated Shal related subfamily D, member 1	KCND1
	potassium channel, voltage gated Shal related subfamily D, member 3	KCND3
	potassium channel, voltage gated subfamily A regulatory beta subunit 1	KCNAB1
	potassium channel, voltage gated subfamily E regulatory beta subunit 3	KCNE3
	potassium channel, voltage gated subfamily E regulatory beta subunit 4	KCNE4
	potassium voltage-gated channel, modifier subfamily S, member 3	KCNS3
Sodium Channel	sodium channel, voltage gated, type III beta subunit	SCN3B
	sodium channel, voltage gated, type IX alpha subunit	SCN9A
Solute Carrier	solute carrier family 1 (glial high affinity glutamate transporter), member 2	SLC1A2
	solute carrier family 1 (glial high affinity glutamate transporter), member 3	SLC1A3
	solute carrier family 16 (aromatic amino acid transporter), member 10	SLC16A10
	solute carrier family 17 (acidic sugar transporter), member 5	SLC17A5
	solute carrier family 18 (vesicular monoamine transporter), member 2	SLC18A2
	solute carrier family 2 (facilitated glucose transporter), member 1	SLC2A1
	solute carrier family 2 (facilitated glucose transporter), member 10	SLC2A10
	solute carrier family 2 (facilitated glucose transporter), member 13	SLC2A13
	solute carrier family 2 (facilitated glucose transporter), member 8	SLC2A8
	solute carrier family 20 (phosphate transporter), member 2	SLC20A2
	solute carrier family 22 (organic cation transporter), member 3	SLC22A3
	solute carrier family 22 (organic cation/carnitine transporter), member 16	SLC22A16
	solute carrier family 22 (organic cation/zwitterion transporter), member 4	SLC22A4
	solute carrier family 22, member 17	SLC22A17
	solute carrier family 24 (sodium/potassium/calcium exchanger), member 1	SLC24A1

solute carrier family 24 (sodium/potassium/calcium exchanger), member 3	SLC24A3
solute carrier family 24 (sodium/potassium/calcium exchanger), member 4	SLC24A4
solute carrier family 25 (mitochondrial carrier; citrate transporter), member 1	SLC25A1
solute carrier family 26 (anion exchanger), member 2	SLC26A2
solute carrier family 26 (anion exchanger), member 7	SLC26A7
solute carrier family 27 (fatty acid transporter), member 1	SLC27A1
solute carrier family 27 (fatty acid transporter), member 2	SLC27A2
solute carrier family 27 (fatty acid transporter), member 3	SLC27A3
solute carrier family 27 (fatty acid transporter), member 4	SLC27A4
solute carrier family 29 (equilibrative nucleoside transporter), member 3	SLC29A3
solute carrier family 30 (zinc transporter), member 1	SLC30A1
solute carrier family 30 (zinc transporter), member 7	SLC30A7
solute carrier family 31 (copper transporter), member 2	SLC31A2
solute carrier family 33 (acetyl-CoA transporter), member 1	SLC33A1
solute carrier family 35 (UDP-GlcNAc/UDP-glucose transporter), member D2	SLC35D2
solute carrier family 35, member A4	SLC35A4
solute carrier family 35, member D3	SLC35D3
solute carrier family 35, member E1	SLC35E1
solute carrier family 37, member 3	SLC37A3
solute carrier family 38, member 6	SLC38A6
solute carrier family 39 (zinc transporter), member 6	SLC39A6
solute carrier family 4 (anion exchanger), member 1, adaptor protein	SLC4A1AP
solute carrier family 4 (sodium bicarbonate cotransporter), member 4	SLC4A4
solute carrier family 4, sodium borate transporter, member 11	SLC4A11
solute carrier family 40 (iron-regulated transporter), member 1	SLC40A1
solute carrier family 5 (sodium/myo-inositol cotransporter), member 3	SLC5A3
solute carrier family 6 (neurotransmitter transporter), member 8	SLC6A8
solute carrier family 7 (cationic amino acid transporter, y+ system), member 2	SLC7A2
solute carrier family 8 (sodium/calcium exchanger), member 1	SLC8A1
solute carrier family 9, subfamily A (NHE3, cation proton antiporter 3), member 3 regulator 1	SLC9A3R1
solute carrier family 9, subfamily A (NHE9, cation proton antiporter 9), member 9	SLC9A9
solute carrier organic anion transporter family, member 1A2	SLCO1A2
solute carrier organic anion transporter family, member 1B3	SLCO1B3
solute carrier organic anion transporter family, member 2A1	SLCO2A1
solute carrier organic anion transporter family, member 2B1	SLCO2B1

Table S5: 103 DE genes in the GSE65135 dataset that belong to the ICT group.

Table S6: DE genes associated with disease stage in the Florence Cohort

gene function	gene name	β Coefficient	p-value	gene type
Cell-matrix adhesion	COL17A1	9,42	0,0037	Collagen Type XVII Alpha 1 Chain
Centriole amplification	CCDC67	15,28	0,0011	Deuterosome Assembly Protein 1
Regulation of transcription	SBNO1	3,92	0,0090	strawberry notch homolog 1
	ZNF673	6,49	0,0055	KRAB box domain containing 4
	HCFC2	3,68	0,0062	host cell factor C2
**gene function**	**gene name**	**β Coefficient**	**p-value**	**gene type**
Arginine and proline metabolism	ADC	-5,74	0,0100	arginine decarboxylase
Cell differentiation	DAZL	-9,19	0,0096	deleted in azoospermia like
	UNC45B	-13,16	0,0088	Unc-45 Myosin Chaperone B
	K6IRS2	-2,25	0,0060	keratin 72
	TMPIT	-4,07	0,0056	Transmembrane Protein 120°
Cell motility	C6orf206	-3,80	0,0036	radial spoke head 9 homolog
Cytoskeleton organization	FGD3	-5,04	0,0047	FYVE, RhoGEF and PH domain containing 3
	JAM3	-10,56	0,0007	junctional Adhesion Molecule 3
ER to Golgi apparatus trafficking	TTC15	-6,41	0,0016	Trafficking Protein Particle Complex 12
GTP binding	LANCL2	-5,46	0,0069	LanC Like 2
Immune response	MAP4K2	-3,56	0,0095	mitogen-activated protein kinase kinase kinase kinase 2
	LRRC7	-23,32	0,0034	leucine Rich Repeat Containing 7
Lipid transport	SPNS3	-2,46	0,0082	sphingolipid transporter 3
Metabolism of proteins	ALG12	-6,88	0,0077	alpha-1,6-mannosyltransferase
Regulation of G-protein coupled receptor [illegible]	USP20	-2,14	0,0070	Ubiquitin Specific Peptidase 20
Regulation of GTPase activity	RAB3IL1	-5,48	0,0008	RAB3A Interacting Protein Like 1
Regulation of protein ubiquitination	C21orf1	-2,79	0,0089	PTTG1 Interacting Protein
Regulation of transcription	AHRR	-25,45	0,0019	aryl-hydrocarbon receptor repressor
	TAF13	-27,29	0,0054	TATA-Box Binding Protein Associated Factor 13
Solute carrier	SLC16A10	-33,59	0,0024	family 16 (aromatic amino acid transporter), member [illegible]
TGF beta receptor signaling pathway	PARD3	-17,79	0,0007	par-3 family cell polarity regulator
unknown	C20orf112	-8,78	0,0057	chromosome 20 open reading frame

Table S6: DE genes associated with disease stage in the Florence Cohort. A Generalized linear model was performed and the β Coefficient was calculated to evaluate the association of the gene expression level with the disease stage. The reported genes show a significant nominal p-value, although they did not pass the canonical threshold ($p<0,05$) after the multiple test correction. In the upper table are reported enes with a positive association, in the lower genes with a negative association.

Table S7: DE-ICT genes involved in the β Oxidation process in DLBCL primary samples from the GSE12195 dataset.

Gene.symbol	Log2FC	adj.P.Val	Gene.title
ACAD10	0.99	0.0008	acyl-CoA dehydrogenase family member 10
ACAD8	0.77	0.0056	acyl-CoA dehydrogenase family member 8
ECHS1	0.82	0.0009	enoyl-CoA hydratase, short chain 1
HADHB	0.49	0.0157	hydroxyacyl-CoA dehydrogenase/3-ketoacyl-CoA; thiolase/enoyl-CoA; hydratase; (trifunctional protein), beta subunit

Table S7: DE-ICT genes involved in the β Oxidation process in DLBCL primary samples from the GSE12195 dataset compared with a cohort of 5 healthy centrocytes present in the same dataset.

Genes reported result deregulated, despite the fact that don't reach the threshold of log2 Fold Change >2 or <-2, but still present a nominal p-value.

Table S8: Expression of the nuclear receptor corepressor 1 in DLBCL primary samples from the GSE12195 dataset.

**Gene.symbol**	**Probe ID**	**Log2FC**	**adj.P.Val**	**Gene.title**
NCOR1	200856_x_at	-0.641078873239433	0.0149266507506678	nuclear receptor corepressor 1
NCOR1	200854_at	-0.739115492957749	0.00784573080872583	nuclear receptor corepressor 1
NCOR1	200855_at	-0.618315492957743	0.0102714665538469	nuclear receptor corepressor 1

Table S8: Expression of the nuclear receptor corepressor 1 in DLBCL primary samples from the GSE12195 dataset compared with a cohort of 5 healthy centrocytes present in the same dataset.

The *NCoR1* gene is recognized by different probes in the dataset, all indicating a down regulation of the gene, despite the fact that its differential expression doesn’t reach the threshold of log2 Fold Change >2 or <-2, but still presents a nominal p-value.
